# Pediatric Patients with Intravascular Devices: Polymicrobial Bloodstream Infections and Risk Factors

**DOI:** 10.4061/2011/826169

**Published:** 2011-04-18

**Authors:** Wes Onland, Dasja Pajkrt, Cathy Shin, Stana Fustar, Teresa Rushing, Wing-Yen Wong

**Affiliations:** ^1^Department of Pediatrics, Emma Childrens Hospital, Academic Medical Center, P.O. Box 22700, 1100 DD Amsterdam, The Netherlands; ^2^Division of Pediatric Surgery, Childrens Hospital Los Angeles, Los Angeles, CA 90027, USA; ^3^Division of Hematology-Oncology, Childrens Hospital Los Angeles, Los Angeles, CA 90027, USA; ^4^Division of Pharmacy, Childrens Hospital Los Angeles, Los Angeles, CA 90027, USA

## Abstract

A retrospective study was conducted, including 61 patients with long-term intravascular devices (IVDs) admitted to the Childrens Hospital Los Angeles with diverse underlying diseases, different types of catheters, and culture-proven catheter-related bloodstream infections (BSIs). Within these patients, 125 catheter-related BSIs occurred, and the incidence of monomicrobial and polymicrobial BSIs was evaluated. Risk factors for polymicrobial BSIs were determined. Forty-two BSIs contained more than one pathogen. These polymicrobial BSIs were observed more often in younger patients (<4.1 years versus ≥4.1 years) and less in patients using venous implanted ports. No other associations were found between the occurrences of polymicrobial BSIs and underlying diseases, other types of catheters, host defense status, parenteral nutrition, recurrences, or catheter removal. Patients with long-term IVDs at a younger age have a higher risk of developing a polymicrobial BSI. Future prospective studies should address the issue of polymicrobial infection in IVDs in more detail.

## 1. Introduction

Intravascular devices (IVDs) are a crucial tool in the treatment of pediatric patients with diverse underlying diseases. They provide a reliable access site for frequent transfusions of blood products, prolonged intravenous medications, chemotherapy, apheresis, parenteral nutrition, and blood sampling. However, catheter-related bloodstream infections (BSIs) have emerged as the most important cause of infections in children with intravascular devices (IVDs) [1]. More than 80% of primary bacteriaemia are considered to be catheter associated in the adult population [2]. 

The incidence of individual monomicrobial pathogens in BSIs have been studied identifying different risk factors, such as age, underlying disease, type of catheter, and immune status [3–6]. However, epidemiological data about the incidence and risk factors of catheter-related BSIs caused by polymicrobial infections are scarce [7]. Two reports in the 1980s and one study most recently describe polymicrobial bacteriaemia in children [8–10], identifying predisposing factors as underlying disease, types of IVD, and immune status. Age of the patients as a risk factor was not included. Recently, catheter-related BSIs were investigated in pediatric patients in ambulatory care, showing that younger children were at a greater risk of obtaining a polymicrobial infection than older children [11]. Since studies in adults have shown that polymicrobial infections are related to higher risks of mortality and morbidity, this association could be of clinical importance [12, 13].

Recently, we have reported a retrospective study on the use of the ethanol-lock technique for the treatment of catheter-related BSIs [14]. In a secondary analysis of this study, we evaluated the frequency of monomicrobial and polymicrobial BSIs in this case series of pediatric patients, and identified the clinical risk factors associated with the occurrence of polymicrobial infections in an inpatient setting.

## 2. Material and Methods

### 2.1. Study Design

Since the primary purpose of the original study was to evaluate the effect of the ethanol-lock technique, patients with catheter-related BSIs in need for the ethanol-lock technique were identified in retrospect by assessing the pharmacy dispensing records. All patients with IVDs treated with ethanol-locks from the 1st of June 2004 through the 22nd of June 2005 were included [14]. Patients were eligible for ethanol-lock treatment if they had persistent positive blood cultures (persistent positive blood cultures after 48-hours' administration of appropriate intravenous antimicrobial therapy), or incidence of multiple catheter-related BSIs. The patient also had to be older than 6 months of age, a patent lumen, and a negative history of allergy to alcohol. These eligible patients were identified after appropriate approval from the CHLA Institutional Review Board. Patients who received ethanol-locks for any other purpose than a catheter-related BSI were excluded. 

Data of all BSIs occurring in the included IVDs were extracted by chart review from the beginning of the study (or the first day of insertion) until the end of the study period, regardless whether the BSIs occurring in that IVD were treated with ethanol-locks. For example, if an IVD was treated with ethanol-lock therapy only during a first BSI but not on the consecutive occasions, the data on the consecutive BSIs in that same IVD during the study period were also included.

### 2.2. Data Extraction and Study Definition

Catheter-related BSIs were defined as clinical suspicion of infection (fever, chills, unexplained leukocytosis, hypotension, and/or tachycardia) with no clear focus apart from the IVD, or signs of local infection around the insertion site, in combination with a positive blood culture from a catheter segment. No concomitant peripheral blood cultures were obtained. 

The collected data included the number of BSIs within the same IVD, the presence of polymicrobial or monomicrobial BSIs, the different pathogens causing the catheter-related BSI, recurrence, and removal of the IVD as a direct result of an infection. The clinical information that was extracted included: age, gender, underlying disease, type of catheter, the use of parenteral nutrition, and the presence or absence of neutropenia. 

A polymicrobial BSI was defined as the isolation of more than one pathogen from the same blood sample or from two consecutive samples within 24 hours. We chose the calculated median age of our case series, as a more objective measurement to divide the patients into a younger and older age group. Patients with diverse underlying diagnoses were divided into four categories, because of the differences in treatment regimens, product exposure and interventions. The first category included oncology patients exposed to chemotherapy, the second consisted transplanted patients because of the intensity of the chemotherapy and the third group included patients with gastrointestinal disorders on long-term parenteral nutrition. The 4th group was defined as nonimmunosuppressed patients with intermittent manipulation of the catheter and a relative short dwelling time. The types of catheters were divided according to their number of lumina. A Broviac is a single-lumen catheter, whereas a Hickmann and Med-comp are double-lumen catheters. Although a venous implanted port is a single-lumen IVD, this device was categorized separately, because it is totally implanted. The PICC (peripherally inserted central catheter) was analyzed separately as well, because this is a nontunneled single-lumen IVD. Neutropenia was defined as an absolute granulocyte count below 500 cells/mm^3^. The usage of long-term parenteral nutrition was defined as dependency on parenteral nutrition for more than 28 consecutive days in one IVD [10]. Since there is no definition available in literature, we defined a recurrent BSI as having another catheter-related BSI within 30 days of the previous one with the same pathogen.

To explore the possible bias of analyzing polymicrobial versus monomicrobial BSIs in IVDs with multiple consecutive BSIs, where the next BSIs might not be independent of the previous one, a subgroup of only the first BSIs in every IVD was analyzed. The usage of ethanol-locks and antibiotics in the previous BSI might also influence the results. Since neither of the primary BSIs in every catheter was treated with this technique or antibiotics, this created subgroup also explored the possible bias of using ethanol-locks and antibiotics.

### 2.3. Statistical Analysis

Comparison of the frequency distribution of polymicrobial BSIs in relation to the different risk factors and outcome were calculated in univariate analyses using odds ratios with 95% confidence intervals (CI) and corresponding *P* values. Multivariate logistic regression models were used to find the determinants independently associated with polymicrobial BSIs in catheter-related infections. All statistical tests were two-sided and considered significant when *P* < .05. All data were analyzed using Intercooled Stata 9.2 (Stata Corporation, College Station, TX, USA).

## 3. Results

In the study period, the pharmacy dispensed ethanol-lock treatments to 79 IVDs in 75 patients. Eleven patients were excluded because the prescribed treatment was for a reason other than infection, such as mechanical problems with the line (*n* = 8), prophylaxis (*n* = 2) or fever of viral origin (*n* = 1). Three patients were excluded due to insufficient data. Compared to our original study fewer patients and BSIs were excluded from the analyses, since the efficacy of the ethanol-locks was not the issue in the present study. Therefore, those patients and events with incomplete indwelling time or period of the ethanol-locks, still on ethanol therapy at the end of the study period and the patients with a PICC inserted could be included in this study [14]. 

The patients' characteristics are shown in Table 1. Four patients received a second IVD during the study period. Sixty-one patients had 125 BSIs during the study period. Median age of the cohort was 4.1 years. There were 4 diagnostic groups; the oncology group consisted of patients with solid tumors or hematological malignancies, the transplant group consisted of patients who were having bone marrow transplantations, as well as recipients of organ transplantation. The gastrointestinal group had patients with short bowel syndrome, pseudo-obstruction syndrome or microvillous inclusion syndrome. The 4th category was a diverse group of patients with benign hematological diagnoses, metabolic disorders, cardiac issues and a patient with VACTERL (vertebral anomalies, anal atresia, cardiac malformations, tracheoesophageal fistula, renal anomalies, and limb anomalies). 

Table 2 lists the pathogens found in the 125 BSIs containing monomicrobial and polymicrobial infections. A total of 186 pathogens were cultured in 42 polymicrobial and 83 monomicrobial BSIs. The polymicrobial BSIs contained relatively more enterococci (*n* = 21). Seventy-four percent of the polymicrobial BSIs contained one or more gram-positive bacilli, 57.1% of the BSIs contained one or more gram-negative BSIs and 11.9% one or more yeast. The monomicrobial BSIs were predominantly Gram-positive BSIs (72.3%).

Results of the univariate analysis of risk factors for a polymicrobial BSI are shown in Table 3. Polymicrobial BSIs were found in 74% of BSIs from children <4.1 years of age compared to only 26% of BSIs from the older age group, with a statistically significant odds ratio. No association was seen between the frequency of polymicrobial BSIs and the different categories of diagnosis or the types of catheter, except for venous implanted ports. Less polymicrobial BSIs were seen in the venous implanted port with a statistical significant odds ratio. Additional analyses combining the PICCs and all single-lumen central catheters, including the venous implanted ports as one group did not reveal a significant association (odds ratio 0.84; 95% confidence interval 0.40, 1.78; *P* = .66). Neither neutropenia nor the usage of long-term parenteral nutrition was associated with a higher risk for polymicrobial BSIs. Polymicrobial BSIs were not associated with a higher risk of recurrence or requiring removal of the affected IVD. 

Analysis of the subgroup including only the first BSI in every IVD, revealed that the age group <4.1 years had 15 polymicrobial BSIs (48.4%) and 16 monomicrobial BSIs (51.6%) compared to 8 polymicrobial BSIs (26.7%) and 22 monomicrobial BSIs (73.3%) in those ≥4.1 years. There was a nonsignificant trend towards an association between the 2 age groups (odds ratio 0.33; 95% confidence interval 0.13, 1.13; *P* = .08).

The multivariate model, including all predefined risk factors, failed due to collinearity and small numbers. However, potential confounders for the association between polymicrobial BSIs and younger age were explored by performing multivariate analyses including one confounder at the time in the model. None of the potential confounders investigated altered the relation between age and the risk of polymicrobial BSIs (Table 4).

## 4. Discussion

In our retrospective study, we found comparable numbers of various bacteria as reported by others [11, 15, 16], but also a considerable amount of polymicrobial BSIs in children with an IVD. A possible explanation for this amount of polymicrobial BSIs could be that only patients treated with ethanol-locks were included in this study. To be eligible for this treatment the catheter-related BSI had to be persistent or recurrent. As recently reported polymicrobial BSIs are associated with prolonged bloodstream infections [10].

The analysis of this study on possible risk factors for polymicrobial BSIs revealed a significantly higher risk of polymicrobial BSI occurrence in younger children. This predominance of polymicrobial BSIs in younger patients has, most recently, been reported in children in an ambulatory setting [11]. In line with this study, we confirm this association in children in an inpatient setting. In addition to this study, we have shown that other clinical factors, such as diverse underlying diagnosis, immune status and the use of parenteral nutrition revealed no such association. The multivariate analysis suggested that the effect of the patient's age on the risk of having polymicrobial BSIs was not biased by any of the other clinical factors included in this study. This finding might have clinical consequences, since mortality and morbidity is higher in polymicrobial infections than in single gram-positive and gram-negative bacteriaemia [12, 13, 17]. We found no relation between the occurrence of polymicrobial BSIs and short-term outcomes as necessitation of removing the IVD or the recurrence of the same pathogens in that catheter. However, since this study was not designed to evaluate the outcome of the occurrence of polymicrobial BSIs, future larger studies should address this topic. 

The second conclusion from this study was that venous implanted ports have a lower risk of getting infected by a polymicrobial BSI than partially implanted catheters. Previous studies had already shown this, leading to a recommendation of using ports, when feasible [18–20]. Our data support that conclusion. 

As described above, a subsequent new BSI in an IVD might not be independent from the previous one in that same catheter and may bias the results described above. Second, the primary choice of antibiotics might be a confounder of the reported results, although hospital policy prescribed the same broad-spectrum antibiotics to all patients, regardless of age. Third, treatments with ethanol-locks might also influence the occurrences of microorganisms in IVDs. We investigated the possible bias of the used antibiotics and ethanol-lock treatment by analyzing only the first BSI of every catheter. Of course, the first infection in every catheter did not have a previous BSI and were not yet treated with the ethanol-locks. This analysis showed the same increased risk of polymicrobial BSIs at a younger age with a higher confidence interval, due to lack of power. This suggested that the inclusion of multiple BSIs of one IVD, and the treatment of some BSIs with ethanol-locks, did not bias the results, thus supporting the analysis of the whole case series. 

This study has several limitations. First, since this was a secondary analysis on a previously reported study on ethanol-locks, there might be a selection bias including patients treated with ethanol-locks. However, we found the same association as the solely written publication on this topic designed to investigate this topic [11]. Together with our analysis including only the first BSI in every catheter, we feel that the effect of selection bias on the found association is less likely.

Second, no concomitant, peripherally drawn blood cultures were obtained as our standard of practice in order to minimize venipunctures in these children with significant venous access difficulties. A distinction between a BSI and contamination might be difficult when skin microbiota is isolated [21]. Gram-positive organisms, especially coagulase-negative staphylococci are judged more to be contaminants than gram-negatives in polymicrobial BSIs in pediatric patients [9]. In our case series, all patients had clinical symptoms of bacteriaemia, and therefore had a high *a priori *chance of having a true BSI. Furthermore, a low frequency of coagulase-negative staphylococci was found in the polymicrobial BSIs, compared to the monomicrobial BSIs. Furthermore, to be eligible for the ethanol-lock treatment and included in this secondary analysis the catheter-related BSI had to be persistent or recurrent, which suggests that these BSI are not likely to be contaminated.

A possible explanation for the association between polymicrobial BSIs and age could be different groups of underlying illnesses with different treatments, immune status and different types of catheters. However, none of the other variables, (with the exception of venous implanted ports) showed a relation with the frequency of polymicrobial BSIs. Furthermore, younger children are less aware of the vulnerability of their IVD, resulting in risky behavior. Younger children also have different skin microbiota with faecal contamination, which might result in a higher risk of getting infected by a polymicrobial BSI than older children [1]. More parameters are to be considered as a risk factor, like the number of manipulations, the site of insertion of the IVDs or the occurrence of chemotherapy-induced mucositis. The number of manipulations represents the most important risk factor for the development of BSI [19]. However, these data were not available in this retrospective study. 

 In conclusion, given the limitations of a retrospective study, our results suggest that a higher risk of polymicrobial BSIs is associated with younger age and lower with venous implanted ports. Future prospective studies including all infants with IVDs, not only those treated with ethanol-locks should confirm these findings and address the issue of polymicrobial infections in IVDs in more detail in view of the risk and difficulty of eradication of these pathogens as well as the need to retain functional IVDs in this vulnerable pediatric population.

##  Conflict of Interests 

The authors declared no conflict of interests.

## Figures and Tables

**Table 1:** Patient characteristics.

	Median (IQR^a^)
Age patients in years	4.1 (1.9–12.5)
	Total number (%)
Gender: male/female	37/24 (60.6/39.4)
Diagnosis	
Oncology	31 (50.7)
BMT/posttransplant	9 (14.8)
Gastroenterology	12 (19.7)
Other^b^	9 (14.8)
Type of catheter	
Broviac single-lumen	13 (20)
Hickman/Med-comp double-lumen	32 (49.2)
Venous implanted port	11 (16.9)
PICC^c^	9 (13.9)
Number of BSIs^d^	
1 BSI	30 (49.2)
2 BSIs	15 (24.6)
3 BSIs	10 (16.4)
4 BSIs	4 (6.6)
6 BSIs	1 (1.6)
13 BSIs	1 (1.6)

^a^IQR: interquartile range; ^b^PICC: peripherally inserted central catheter; ^c^including benign hematological diagnosis, metabolic disorders, cardiac issues, and a patient with VACTERL; ^d^BSI: catheter-related bloodstream infection.

**Table 2:** Monomicrobial and polymicrobial BSIs.

Pathogens	Monomicrobial BSI (%)	Polymicrobial BSI (%)^a^	Total (%)
Gram-positive bacteria	60 (72.3)	53 (51.5)	113 (60.8)
a-hemolytic streptococci (viridans)	1	2	
*Streptococcus pneumoniae*	—	1	
Coagulase-negative staphylococci	42	21	
*Staphylococcus aureus*	3	4	
Methicillin-resistant *Staphylococcus aureus *	3	1	
*Enterococcus *sp.	7	21	
Bacillus sp.	4	3	
Gram-negative bacteria	15 (18.1)	44 (42.7)	59 (31.7)
*Enterobacter Klebsiella Serratia *group	9	20	
*Escherichia coli*	1	3	
*Pseudomonas* sp.	1	8	
*Stenotrophomonas maltophilia*	1	3	
*Actinobacter *sp.	3	5	
Other Gram negatives	—	5	
Yeast	8 (9.6)	6 (5.8)	14 (7.5)
*Candida albicans*	1	—	
*Candida* non*albicans *	7	6	

Total	83 (100)	103 (100)	186 (100)

^a^Total of 103 pathogens in 42 polymicrobial BSIs (33.6% of 125 BSIs).

**Table 3:** Associations between polymicrobial/monomicrobial BSIs and the different risk factors and outcome.

	Monomicrobial BSIs^a^ (%)	Polymicrobial BSIs (%)	Odds ratio	95% CI^b^	*P* value
Age					
<4.1	40 (48.2)	31 (73.8)	1.0^c^		
≥4.1	43 (51.8)	11 (26.2)	0.33	0.15–0.74	.007*
Diagnosis					
Oncology	34 (41.0)	21 (50.0)	1.0^c^		
Transplantation	22 (26.5)	6 (14.3)	0.44	0.15–1.27	.13
Gastrointestinal	16 (19.3)	10 (23.8)	1.01	0.39–2.64	.98
Other	11 (13.2)	5 (11.9)	0.74	0.22–2.42	.61
Type of catheter					
Single lumen	22 (26.5)	16 (38.1)	1.0^c^		
Double lumen	38 (45.8)	21 (50.0)	0.76	0.33–1.75	.52
Venous implanted port	18 (21.7)	3 (7.1)	0.23	0.06–0.91	.038*
PICC^d^	5 (6.0)	2 (4.8)	0.55	0.09–3.20	.51
Neutropenia					
No	69 (83.1)	34 (81.0)	1.0^c^		
Yes	14 (16.9)	8 (19.0)	1.16	0.44–3.03	.76
Parenteral nutrition					
No	58 (69.9)	27 (64.3)	1.0^c^		
Yes	25 (30.1)	15 (35.7)	1.29	0.59–2.83	.53
Recurrence					
No	28 (80.0)	20 (76.9)	1.0^c^		
Yes	7 (20.0)	6 (23.1)	1.20	0.35–4.11	.77
Removal					
No	63 (75.9)	33 (78.6)	1.0^c^		
Yes	20 (24.1)	9 (21.4)	0.86	0.35–2.10	.74

^a^BSIs: blood stream infections; ^b^95% CI: 95% confidence interval; ^c^reference group; ^d^PICC: peripherally inserted central catheter; *statistical significant.

**Table 4:** Odds ratios for the relation between polymicrobial BSI and age of patients adjusted for potential clinical confounders.

Potential confounder	Adjusted OR^a^ (95% CI^b^)	*P* value
Diagnosis	0.29 (0.13, 0.68)	.004
Type of catheter	0.39 (0.16, 0.95)	.038
Neutropenia	0.33 (0.14, 0.74)	.008
Parenteral nutrition	0.33 (0.14, 0.77)	.009
Recurrence	0.25 (0.08, 0.82)	.023
Removal	0.33 (0.14, 0.75)	.008

^a^OR: odds ratio; ^b^95% CI: 95% confidence interval.
